# Should Preachers Pay?

**Published:** 1887-03

**Authors:** 


### SHOULD PREACHERS PAY?

### A DANIEL COMES TO JUDGMENT.

*Editors Atlanta Medical and Surgical Journal:*

Your correspondent, who hails from Rome, Ga., and discusses in your January number the above-stated question, is evidently both self-reliant and aggressive.

He concedes in the opening sentence of his essay that universal custom, and he might have added immemorial usage, are against charging ministers of the gospel for medical services. He justly fastens the blame and responsibility of this usage, which he characterizes as "abominable," not on the clergy, but on his own guild.

Against the unbroken consensus of professional opinion he files his modest protests, backed by a group of alleged facts that are as unreal as the mockery of a vision, or the vague fancies of a sick man's dream.

With a measure of self-complacency that is simply marvelous, he straightway proceeds to urge its abrogation as alike dishonoring to the clergyman and damaging to the physician.

It is somewhat singular that it did not occur to your astute contributor that time-honored and well-nigh universal customs, like the one he so defiantly challenges, are quite sure to rest on a substratum of common sense and obvious propriety that it is not altogether prudent to antagonize. Especially is this true when it is borne in mind that this particular usage has had the sanction and even the emphatic endorsement of all the greater lights of the medical profession.

It is fair to admit that he allows for what he styles "exceptional cases," and that he does not wholly exclude the idea of charity from professional practice. This speaks volumes for his heart, but heavily discounts his head. Indeed, these concessions, while they save him from the horn of a dilemma, are manifestly suicidal, so far as his main argument is concerned.

The mistake of your contributor is in accepting a literal *quid pro quo* theory as a sufficient basis both of medical ethics and of professional etiquette. In its last analysis it is the "pound of flesh" which Shylock demanded because it was so nominated in the bond. It is unfortunate, both for him and his argument, that he has overlooked the fact that with scarcely a noteworthy exception there exists an intimate, and to a large extent a confidential, relationship between the clerical and medical professions. They are often brought face to face at the bedside of the diseased and the dying. This of itself constitutes a bond of sympathy between them. In addition to this fact there is this other consideration, that they both perform a great deal of gratuitous work for which they receive neither tangible reward nor even the prospect thereof. The clergyman, not less than the physician, is called to breast the wintry storm and to face deadly malaria that he may minister to the poor and the dependent. The vast majority of physicians, notably John Mason Goode, the great London practitioner, esteem the most lucrative branch of their practice that which is devoted to Christ's poor—not lucrative, it is true, in guineas or greenbacks, but in treasure laid up in heaven.

Your contributor suggests that ministers ought to receive adequate salaries, and intimates that this is the only great obstacle to the successful working of his medical reform. How this adequate increase of salary is to be effected is a problem that presbyteries, associations and conferences have wrestled with to little purpose. Perhaps your contributor can untie or sever this Gordian knot. If so, he will be selected as a public benefactor. The practical inconveniences which he conceives to result to the ministry from the custom he so vigorously combats are, as we have already stated, simply fanciful. We have yet to see the clergyman who has been neglected by his family-physician because he was not technically a pay-patient. Nor is he called to make any compromise of his proper self-respect, or to be in anywise humiliated or fettered by the existing usage. So that your sympathetic contributor may spare himself any vicarious mortification or sense of wounded honor on their behalf.

To be consistent he ought to push his reform to its legitimate logical results. In doing this he will make it obligatory on physicians to charge each other for medical attention. Considered from his own standpoint, this conclusion is inevitable; for is it not true that the average income of physicians greatly exceeds that of ministers? Indeed, the minister's compensation is sometimes less than the wages of an ordinary day-laborer, and oftener than otherwise falls below the income of a skilled mechanic. If your contributor should lack the courage of his convictions, he will most likely hesitate at this point. If so his reform becomes what an evolutionist would call a striking example of "arrested development."

Whilst we cherish feelings of the utmost kindness towards your contributor, we must record our dissent from some awkward phrases and objectionable terms in his article. This applies with special force to the word "mendicant" in connection with a class of men who, all thoughtful men will allow, are precious factors in our Christian civilization. For such a flagrant misuse of our mother tongue, as the only adequate remedy, we would respectfully prescribe a more intimate acquaintance with the English dictionary.

